# Spatial Prediction of N_2_O Emissions in Pasture: A Bayesian Model Averaging Analysis

**DOI:** 10.1371/journal.pone.0065039

**Published:** 2013-06-04

**Authors:** Xiaodong Huang, Peter Grace, Wenbiao Hu, David Rowlings, Kerrie Mengersen

**Affiliations:** 1 Mathematical Sciences, Queensland University of Technology, Brisbane, Australia; 2 Institute of Sustainable Resources, Queensland University of Technology, Brisbane, Australia; 3 School of Population Health, The University of Queensland, Brisbane, Australia; University of Florida, United States of America

## Abstract

Nitrous oxide (N_2_O) is one of the greenhouse gases that can contribute to global warming. Spatial variability of N_2_O can lead to large uncertainties in prediction. However, previous studies have often ignored the spatial dependency to quantify the N_2_O – environmental factors relationships. Few researches have examined the impacts of various spatial correlation structures (e.g. independence, distance-based and neighbourhood based) on spatial prediction of N_2_O emissions. This study aimed to assess the impact of three spatial correlation structures on spatial predictions and calibrate the spatial prediction using Bayesian model averaging (BMA) based on replicated, irregular point-referenced data. The data were measured in 17 chambers randomly placed across a 271 m^2^ field between October 2007 and September 2008 in the southeast of Australia. We used a Bayesian geostatistical model and a Bayesian spatial conditional autoregressive (CAR) model to investigate and accommodate spatial dependency, and to estimate the effects of environmental variables on N_2_O emissions across the study site. We compared these with a Bayesian regression model with independent errors. The three approaches resulted in different derived maps of spatial prediction of N_2_O emissions. We found that incorporating spatial dependency in the model not only substantially improved predictions of N_2_O emission from soil, but also better quantified uncertainties of soil parameters in the study. The hybrid model structure obtained by BMA improved the accuracy of spatial prediction of N_2_O emissions across this study region.

## Introduction

Soils have been considered as an important source for nitrous oxide (N_2_O), a well-known greenhouse gas [1]. N_2_O fluxes often exhibit spatial autocorrelation at multiple scales due to the distribution of soil properties and topography. It is difficult to precisely estimate annual N_2_O emissions at a field scale level because of high spatial variability within the field [2]. In light of these large uncertainties in prediction, spatial variation should be an explicit consideration in any analysis of N_2_O emissions [3]–[5].

To date, the relationship between N_2_O emissions and environmental covariates has largely been quantified by aggregating over all sites and assuming independent observations in multiple linear regression models. However, the presence of spatial correlation can render these models invalid since they can lead to biased estimates and incorrect inferences [6], [7].

In the past decade, a variety of models that take into account the spatial nature of data have been developed [8], [9] and are widely applied in ecology, epidemiology, economics and so on. These models can help to better identify and explore influential factors and guide more informed inferences, as well as improve further experimental design in order to obtain more precise estimates [10].

Bayesian spatial conditional autoregressive (CAR) models are appropriate for all locations that have a similar size and are regularly arranged [11], whereas geostatistical models are more suitable for spatial data with unidentified neighbourhoods [7]. Most published research on the comparison of spatial models has been based on areal data with identified neighbours or point data with a regular sampling pattern [7]–[9], [12], [13]. However, differences between the CAR model and geostatistical model with respect to parameter estimation and predicted spatial distribution based on point-referenced data with an irregular sampling interval and undetermined boundaries are not well understood.

One concern with spatial models is that different representations of the spatial correlation based on the same dataset might give different estimated effect sizes, inferences about significant parameters or estimated error structures [7], [9]. Many candidate spatial correlation structures are available in spatial analysis. It is often difficult to determine the best spatial correlation structure based on standard information-based criteria. However, Bayesian model averaging (BMA) can take account of such model uncertainty and provide better average predictive performance [14], [15]. For example, Boone and Bullock [16] used BMA to pool information from four spatial candidate structures in the analysis of a loblolly pine dataset.

In this study, we consider three spatial correlation structures (independence, distance-based and neighbourhood-based) in spatial analyses of N_2_O emission for point data obtained from irregular sampling intervals in pasture. All models are developed under a hierarchical Bayesian inferential framework. Key attributes of Bayesian approaches are the use of probability for quantifying uncertainty in inferences, formal accommodation of parameter uncertainty [17], and flexibility of model description [8], [18]. The deviance information criterion (DIC) is used to compare the various models [19] and provide weights for BMA [20]. The aims of this study are to assess the effects of various spatial dependencies on spatial prediction, to calibrate spatial predictions of N_2_O by BMA across the study region based on the environment-N_2_O relationships obtained from the three models.

## Materials and Methods

### Study Site and Data Collection

The study site is located at Mooloolah (26°38′40′′ S., 152°56′23′′E.) on the Sunshine Coast in Australia. N_2_O (ug N_2_O-N m^−2^ hr^−1^) emissions and 7 potential independent variables (gravimetric soil moisture (%), soil temperature (°C), soil NO_3_^−^ concentration (Kg N ha^−1^), soil pH; soil sand, silt and clay content (%)) were measured at 17 chambers randomly placed across a 271 m^2^ subtropical pasture at monthly intervals between October 2007 and September 2008.

The pasture was a mixture of the tropical grass *Setaria sphacelata* and the legumes Silverleaf Desmodium (*Desmodium uncinatum*) and White Clover (*Trifolium repens*). No nitrogenous fertilizer had been applied to the pasture site for over 20 years. The soil was classified according to the Australian Soil Classification as a Haplic, Eutrophic, Black Dermosol [21] and had a bulk density of 1.0 g cm^−3^ (0–10 cm) and an organic carbon content of 2.8%. Average soil texture across the site was classified as a loam.

The closed static chamber technique was used for measurements of N_2_O emissions. Chambers were 200 mm high (diameter 200 mm) inserted 100 mm into the soil, allowing a headspace of 80–100 mm. Chambers remained in situ throughout the length of the experiment. Chambers were closed for one h and sampled using 12 ml evacuated glass vials (Exetainer; Labco, High Wycombe, Buckinghamshire, UK) at zero (0) min and 60 min. Full details of chamber method and site climate are described in Rowlings et al. [22].

### Statistical Analysis

The observed data can be defined as point-referenced data [23]. In order to assess the effects of different covariance structures on estimates of spatial variation in N_2_O fluxes and compare the estimated parameters among three Bayesian spatial models, we used a Thiessen-polygon approach to convert point-referenced data to areal data. This method creates a polygon enclosing each original point, such that each point has its own polygon. The defined boundaries of the Thiessen polygons can be used to establish a neighbourhood weight matrix for each data point [24]. In this study, we focused on a linear regression model with three different correlation structures: 1) independent model (no spatial correlation structure), 2) geostatistical (EXP) model (spatial correlation described as the exponential decay function of the distance between pairs of points), and 3) conditional autoregressive model (spatial correlation described as first-order neighbourhood). Although other spatial correlation models, for example, simultaneous autoregressive model (SAR) and geostatistical models with other Matérn correlation functions are available, CAR and EXP models are most commonly used in practice [23].

In all of the following models, let *y_ir_* be the observed N_2_O fluxes at location *i* for replicate *r*, (*i* = 1,…,*Q*, *r* = 1,…,*M*; *Q* = 17, *M* = 13). The vector ***Y_i_*** = [*y_i1,_y_i2,_y_i3,_…,y_iM_*] represents N_2_O fluxes at the *i*th location. Let *X_ir_* be a vector of length *K* = 7, representing the covariates comprising soil moisture, soil temperature, NO_3_^−^, soil texture (including sand, silt and clay) and soil pH at the *i*th site for replicate *r*. Measurements of N_2_O fluxes exhibited skewness, so were log-transformed to better approximate a normal distribution.

### Bayesian Linear Regression Model (Independent Structure)

In this first model, we assumed that locations were independent and that N_2_O emissions were affected by the nominated covariates independently at each location so that:
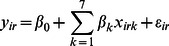
(1)where *β_k_* are the regression coefficients and 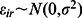 is the residual under the independence and normality assumptions [25]. In a Bayesian framework, the posterior distribution for the parameters of interest is thus given by:



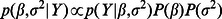
where 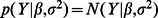. Diffuse priors were imposed for the regression parameters, so that *β* ∼ *N*(0.0, 1.0E6) and σ ∼*U*(0,5).

### Bayesian Geostatistical Model (EXP Model)

The second model considered is an extension of the normal linear regression described above, with an additional term to account for spatial correlation between the experimental sites. The additional term is modelled as a random effect with the variance reflecting the spatial correlation. Letting *s* = (*s_i_*; *i* = 1,…,17) be the vector of site-specific spatial Gaussian random effects, equation (1) is extended as follows:
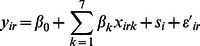
(2)

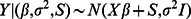


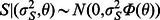


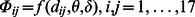


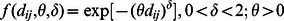



Here, 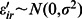 is a spatially uncorrelated error term; *I* is a *r* × *r* identity matrix and 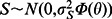 is assumed to be a stationary, isotropic Gaussian process with mean zero and correlation matrix Φ with elements Φ*_ij_* = *ƒ*(*d_ij_,θ,δ*) between *s_i_* and *s_j_* [23]. The pairwise correlations Φ*_ij_* are usually described as a parametric function of the distance *d_ij_* between each pair of sites *i* and *j*. The exponential decay function ƒ(*d_ij_,θ,δ*) = *exp*[−(*θd_ij_*)*^δ^*] [17] is the most popular. Here *θ* is the rate of decrease in spatial correlation per unit of distance, with a large value of *θ* indicating that the spatial correlation decreases rapidly [6]. The prior distribution for *θ* was specified as Uniform with lower and upper bounds corresponding to a correlation of 0.05 the maximum distance (25.35 m) and minimum distance (0.75 m), respectively, between any pair of locations across the study site [26]. Covariate coefficients were modelled with diffuse normal prior distributions *β* ∼ *N*(0.0, 1.0E6). The parameter *δ* controls the amount of spatial smoothing. Thomas et al. [27] advise a value of *δ* = 1. The standard deviation σ was described by a uniform prior σ ∼ *U*(0,5).

### Bayesian Spatial Intrinsic Conditional Autoregressive Model (CAR)

The third model considered employed a different representation of the spatial nature of the data. Here equation (1) is extended as follows:
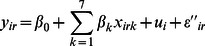
(3)

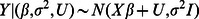


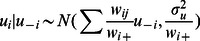
(4)Here 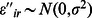 is the within-site residual variation. A conditional autoregressive (CAR) model was used to describe the spatial component. This is represented by the term *U*, with elements *u_i_* denoting the local dependence at site *i* as a function of the site’s neighbours *u_-i_*, where *u_-i_*_ = _[*u_1_*_,_ *u_2_*, …, *u_i−1_*, *u_i+1_*,…,*u_Q_*] [28]. The local neighbourhood relationship is represented as a symmetric *n*×*n* matrix *W* of spatial weights with elements *w_ij_*, and 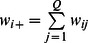. This representation allows a great deal of flexibility in describing the spatial correlation. For example the spatial neighbourhood may be specified only as first-order neighbourhood for each site, in which case *w_ij_* = 1 if sites *i* and *j* share a boundary, and zero otherwise. As before, all covariate coefficients had diffuse normal priors, given by *β* ∼ *N*(0.0, 1.0E6), and σ*_u_* and σ had uniform priors, σ*_u_* ∼*U*(0,10) and σ ∼ *U*(0,5).

### Bayesian Model Averaging (BMA)

Bayesian model averaging can account for model uncertainty by taking a weighted average of models over a given model space [14]. Let *M* be the model space, comprising *L*≥1 model structures *M_l_* with parameter set 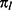
based on data (*D*). Let Δ be the quantity of interest; this could represent, for example, the posterior predictive distribution of *y*. Hence the posterior distribution of Δ given data *D* is [14]:
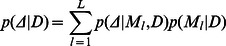



The posterior probability for *M _l_* is given by:
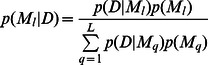
where 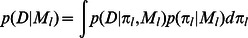.

Here, *p*(*D|M_l_*) is the marginal likelihood of the data *D* given model *M_l_* and *p*(*π_l_ | M_l_*) is the prior density of π*_l_* given model *M_l_*. *p*(*M_l_*) is the prior probability for model *M_l_* when *M_l_* is regarded as the true model [14]. A Laplace approximation, typically the Bayesian information criterion (BIC) [29] can be used to approximate *p*(*D|M_l_*) [14], [30], [31]:
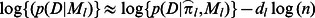


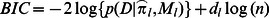
Here 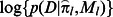 is the maximized log-likelihood of model *l*, which estimates goodness of fit; *d_l_* is the number of parameters in model *l*, and *n* is the sample size. In the absence of other information, it is common to assume equal prior model probabilities *p*(*M_l_* ) for the candidate models [16], [31]. Hence the BMA weights are approximately



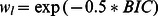



The posterior probability for *M_l_* is calculated as
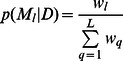



Other information criterion can be used instead of the BIC. For example Akaike’s information criterion 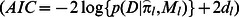 [32] was suggested by Jackson et al. [31]. In the present study, candidate models were compared and combined using the deviance information criterion (DIC) [19]. The DIC is based on the posterior expectation of the deviance 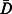 and the effective number of parameters 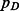 in the model, and is expressed as:
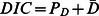



Deviance is defined as 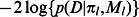 where 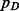 is the difference between the expected deviance and the deviance value for the posterior expectation. The DIC is easily computed from the samples generated through MCMC [23]. A smaller DIC value indicates a better model fit, accounting for model parsimony. In the BMA analysis, we let *p*(*M_l_*) be 1/3, indicating no prior preference for any of the three correlation structures considered in this study.

### Bayesian Analysis and Spatial Interpolation

Markov chain Monte Carlo (MCMC) was used to obtain distributions and corresponding posterior structures of means, standard deviations and quantiles for parameters of interest. Convergence was assessed by checking the trace and the autocorrelation plots for the sample of each chain [33]. For each model we ran a single MCMC chain for 150,000 iterations, discarding the first 50,000 iterations as burn-in. The MCMC analysis was undertaken using WinBUGS software version 1.4 [34].

The posterior predictions of N_2_O obtained from the three models and hybrid model developed by BMA were mapped across the study site using GS+ software [35]. If input values are available across the study site, the model can be used to provide predictions between the experimental locations. In our case, these values were not available, so kriging was used for spatial interpolation of the predicted N_2_O values.

## Results

Summary statistics for observed N_2_O and covariates were provided in Table 1. The overall means were 27.4 ug N_2_O-N m^−2^ hr^−1^, 19.0 Kg N ha^−1^, 35.6%, 22.2°C and 5.5 for N_2_O, NO_3_^−^, soil moisture, soil temperature and soil pH under 17 sampling chambers, respectively. N_2_O tended to be more variable. For soil texture across 17 chambers, the overall means were 18.4%, 44.3% and 37.3%, with range 9.7 to 23.3, 34.4 to 60.9 and 22.8 to 50.9% for soil clay, soil silt and soil sand, respectively. Soil clay had lower percentages in soil texture.

**Table 1:** Summary statistics of observed variables for the 17 chambers over the sampling period from a subtropical pasture at Mooloolah, Queensland.

Variables	Mean	SD	Minimum	Maximum
N_2_O (µg N_2_O-N m**^−^** ^2 ^hr^−1^)	27.2	39.4	0.0	280.4
NO_3_ **^-^** (kg N ha**^−^** ^1^)	18.98	14.1	0.0	90.34
Gravimetric soil moisture (%)	35.57	9.27	12.37	70
Soil temperature (°C)	22.16	3.07	14.8	27.3
pH	5.47	0.29	5.2	6.4
Sand (%)	37.25	7.74	22.75	50.89
Silt (%)	44.34	7.2	34.44	60.94
Clay (%)	18.4	3.07	9.65	23.34

The DIC values, measuring goodness of fit of each model, are shown in Table 2. The DIC was obviously smaller for the CAR and EXP models compared with the independent model, indicating the value of including spatial dependency in describing the N_2_O emissions in this dataset. The DIC values were similar for the CAR model and EXP model, indicating little difference in overall goodness of fit between the two representations of spatial variation. The results also showed that there were 2.7%, 1.8% and 1.4% of observed values that did not fall within the 95% posterior predictive intervals for the linear regression model, CAR and EXP model, respectively. The sum of the squared residuals from the geostatistical model was 317.12, while the sum of the squared residuals from the CAR model was 319.5.

**Table 2:** Posterior means and 95% credible intervals of parameters for three models for pasture.

Parameter	CAR model	EXP model	Independent model
	Mean	Mean	Mean
*β_0_*	−49.2 (−100–6.89)	162.6 (−1728–2049)	−5.84 (−40.62–30.2)
*β_soil moisture_*	0.055 (0.034–0.075)	0.054 (0.034–0.075)	0.039 (0.02–0.06)
*β_soil temperature_*	0.16 (0.1–0.22)	0.16 (0.1–0.22)	0.15 (0.08–0.21)
*β_NO3_^−^*	−0.018 (−0.031– −0.004)	−0.017 (−0.031– −0.003)	−0.006 (−0.02–0.008)
*β_Ph_*	0.4 (−0.73–1.55)	0.32 (−1.19–1.84)	0.21 (−0.42–0.91)
*β_sand_*	0.45 (−0.11–1.0)	−1.67 (−20.54–17.22)	0.023 (−0.35–0.37)
*β_silt_*	0.46 (−0.096–0.978)	−1.66 (−20.52–17.24)	0.033 (−0.34–0.38)
*β_clay_*	0.4 (−0.16–0.94)	−1.7 (−20.57–17.21)	0.004 (−0.37–0.36)
*σ^2^*	1.59 (1.31–1.92)	1.59 (1.30–1.93)	2.0 (1.66–2.43)
*σ^2^_u_*, *σ_s_^2^*	1.78 (0.46–4.81)	0.76 (0.25–1.84)	
*DIC*	745.75	746.1	786.69

Table 2 shows the posterior means and 95% credible intervals (CI) of parameters for the three models. Soil moisture and soil temperature had a substantive positive relationship with N_2_O emissions in all three models. Only the two spatial models showed a negative relationship between N_2_O emissions and NO_3_^−^ in the presence of the other variables in the model. Soil pH and soil texture, such as clay, silt and sand, were not substantial influential factors for N_2_O emissions in the three models in this study.

**Figure 1:**
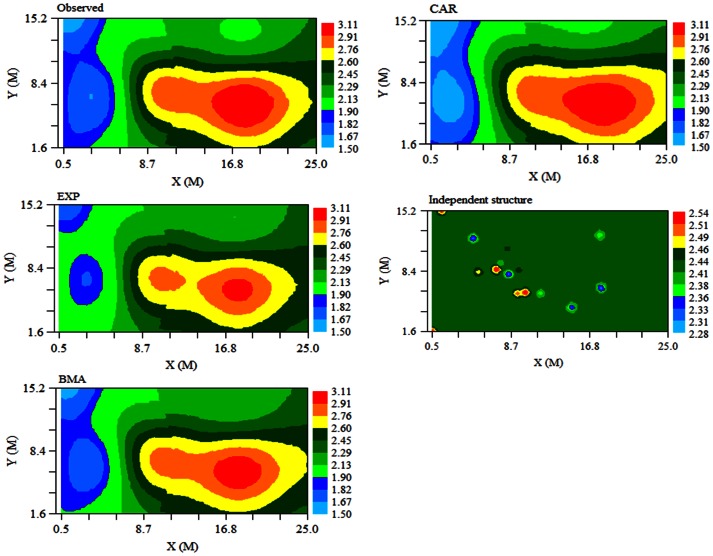
Maps of observed and posterior mean Ln(N_2_O) (ug N_2_O-N m ^−2^ hr ^−1^) from the CAR, EXP, BMA and linear regression models across the study site in pasture.

The spatial patterns of predicted N_2_O using the CAR and EXP models were similar to the observed spatial pattern, particularly the CAR model (Figure 1). However, there were slight errors for classifications of areas into different emission level groups for the two spatial models, particularly the EXP model. The results of the independent model could not match the observed spatial distribution of N_2_O emission.

**Figure 2:**
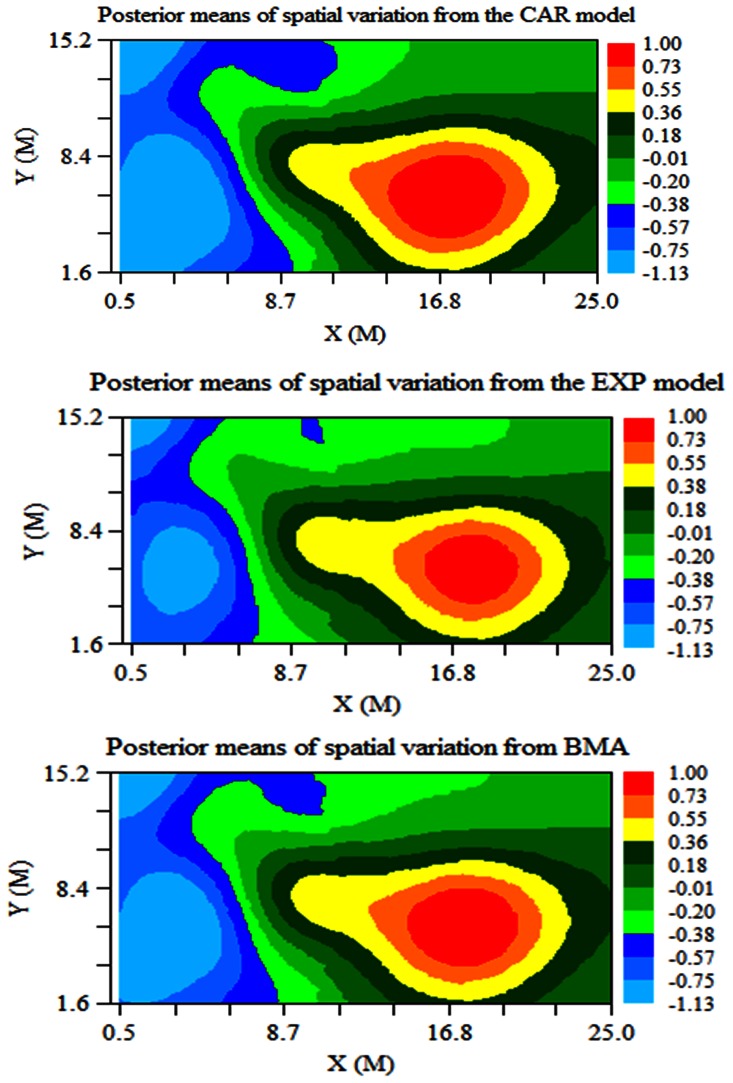
Maps of the posterior means of spatial variation in Ln(N_2_O) (ug N_2_O-N m^−2^ hr^−1^) emission using two spatial models and Bayesian model averaging in pasture.

Figure 2 shows the distributions of the posterior means of the spatial variation in N_2_O emissions which were obtained using the two spatial models and BMA model. The three maps of posterior spatial variation show similar patterns. However, the CAR model displayed slightly larger areas for high or low spatial variation in N_2_O than those from the EXP model (Figure 2).

The CAR model had the highest posterior probability of 5.434E-1, whereas the independent model had a negligible probability of 0.000E-1. The EXP model had a posterior probability of 4.566E-1. The map of the averaged posterior predicted N_2_O emissions across the three structures was much more similar to the map of observed N_2_O emissions comparison to the maps of the CAR, EXP and independent models (Figure 1). The map of averaged spatial predictions of N_2_O displayed better performance in high or low emission areas than that obtained from the EXP model and also it improved the accuracy of the spatial prediction of N_2_O on left side of the map compared with the CAR model. There were also slight changes in the map of the distribution of averaged posterior spatial variation (Figure 2).

## Discussion

The CAR and EXP models are popular approaches for describing spatially correlated data and are widely used in many areas of scientific research. In this case study, we applied these two models and a baseline model that ignored the spatial correlation altogether. In order to gain some insight into the effects of different assumed spatial correlation structures on parameter estimation and spatial prediction of N_2_O emission for the same point data on an irregular grid, and to account for the uncertainty in evaluating spatial variability of N_2_O emissions using Bayesian model averaging.

All three models identified soil temperature and soil moisture as potentially important influential factors positively associated with N_2_O emissions. This is supported by a large body of previous research [5], [36]–[42]. In this study, the average of soil moisture was around 36% in the pasture. Nitrification occurs when soil water-filled pore space is <60% [43]. Our result supported that increasing soil moisture and soil temperature increased N_2_O emissions via the nitrification pathway [36], [39], [44]. Only the CAR and EXP models yielded a significant coefficient for NO_3_^−^.The inverse relationships between N_2_O and NO_3_^-^ from nitrification have been found in grass-clover pasture and laboratory study [36], [45]. The results showed that allowing for spatial dependence in the model affected not only the scale of posterior mean but also affected the determination of significant factors in the model for the pasture data. Moreover, the different spatial correlation structures in the models resulted in differences in the magnitude of the corresponding coefficients. Hence, the selection of an appropriate model structure is a critical step [9].

The sum of the squared residuals showed that the geostatistical model was slightly better than the CAR model. However, the plots of spatial interpolation of the predicted N_2_O values by kriging showed that the geostatistical model tended to oversmooth high N_2_O emission areas in comparison to the results of CAR model. We found that the predicted N_2_O values of the locations which were close to the highest emission site were underestimated by the geostatistical model in comparison to the CAR model and the observed data. The tendency of the EXP model to oversmooth is supported by Best [8]. On the other hand, mapping the spatial prediction of interest is often an important aim of developing a spatial model. Spatial interpolation is a straightforward approach for spatial prediction. The map (Figure 1) based on the CAR model was visually quite similar to the map based on observed data, in that the patch of predicted high emissions under the CAR model matched the observed high emission locations well. The map for the linear regression model indicated that it oversmoothed the study region and poorly predicted the spatial distribution, in that it did not capture some of the regions with low or high emissions. We suggest that the CAR model is better at capturing the distribution of high N_2_O emissions areas in this study.

The posterior spatial variation in N_2_O emissions using the CAR model based on a first-order neighbourhood function tended to be slightly greater than these of the EXP model based on an exponential distance decay function across the study site (Figure 2). This indicated that the CAR model gave more weight to the random effect than did the EXP model. However, the two spatial correlation structures largely revealed similar natural phenomena associated with geographic variation in this study. Finally, the N_2_O distribution could be predicted well across the survey region based on the environmental covariates-N_2_O relationship only, after adjusting for spatial autocorrelation in the models in this study.

Both the CAR model and the EXP model yielded similar parameter estimates for N_2_O emissions underlying point-referenced data with irregular sampling intervals. Our results support previous assertions that the CAR model is comparatively more flexible, and hence potentially more accurate and precise, for such data structures in that it can better represent geographic phenomena and accommodate more complex spatial structures [9]. Finally, the computing time of the CAR model was much faster than that of geostatistical model due to the different representations of the weight matrices [6].

The best spatial correlation structure was unclear based on the DIC values associated with the CAR and EXP models in this study. The two spatial models showed differences in spatial prediction of N_2_O distribution. This justified model averaging across the three structures via BMA. In this article, the posterior model probability was approximated by the DIC, which can be considered as a Bayesian analogue of the AIC suitable for hierarchical models with random effects [19]. Jackson et al. [31] suggested that it was worth investigating the use of the DIC as a basis for model averaging, given the increasing popularity of Bayesian hierarchical models. Our results clearly indicated that the spatial prediction of N_2_O from the hybrid structure could better capture the observed N_2_O distribution across the study region than any of the individual component models. We therefore concluded that Bayesian model averaging was a potentially useful method to take account of uncertainty of different spatial correlation structures and could improve the accuracy of spatial prediction of N_2_O emissions.

In this research, it is acknowledged that spatial analysis not only improves prediction but also highlights clustering and probabilistic uncertainties. The maps of the spatial distribution and the spatial variation in N_2_O emissions may help to guide cultivation practices and determine emission reduction strategies. More attention should still be paid to how to select appropriate spatial correlation structure to improve the accuracy and precision of spatial prediction of N_2_O in study regions such as the one described here.

### Conclusions

Our study showed that soil temperature, soil moisture and NO_3_^-^ were important influential factors in N_2_O emissions in pasture across this study region. High emission areas were accompanied by high uncertainties after taking soil moisture, soil temperature, NO_3_^-^, soil pH and soil texture into account. It was important to incorporate spatial dependency in the model when quantifying the relationship between N_2_O emissions and environmental factors for this pasture. Allowing for spatial dependency in the model could yield accurate spatial prediction of N_2_O. The CAR and EXP models yielded similar parameter estimates based on point-referenced data with irregular sampling intervals. The CAR model was better at capturing high N_2_O emissions areas. The hybrid model structure obtained by BMA could improve the accuracy of mapping the spatial prediction of N_2_O emissions in pasture across this study region. The maps of spatial distribution and spatial variation in N_2_O can improve subsequent experiment design and investigate unknown influential environmental covariates in a study site.
